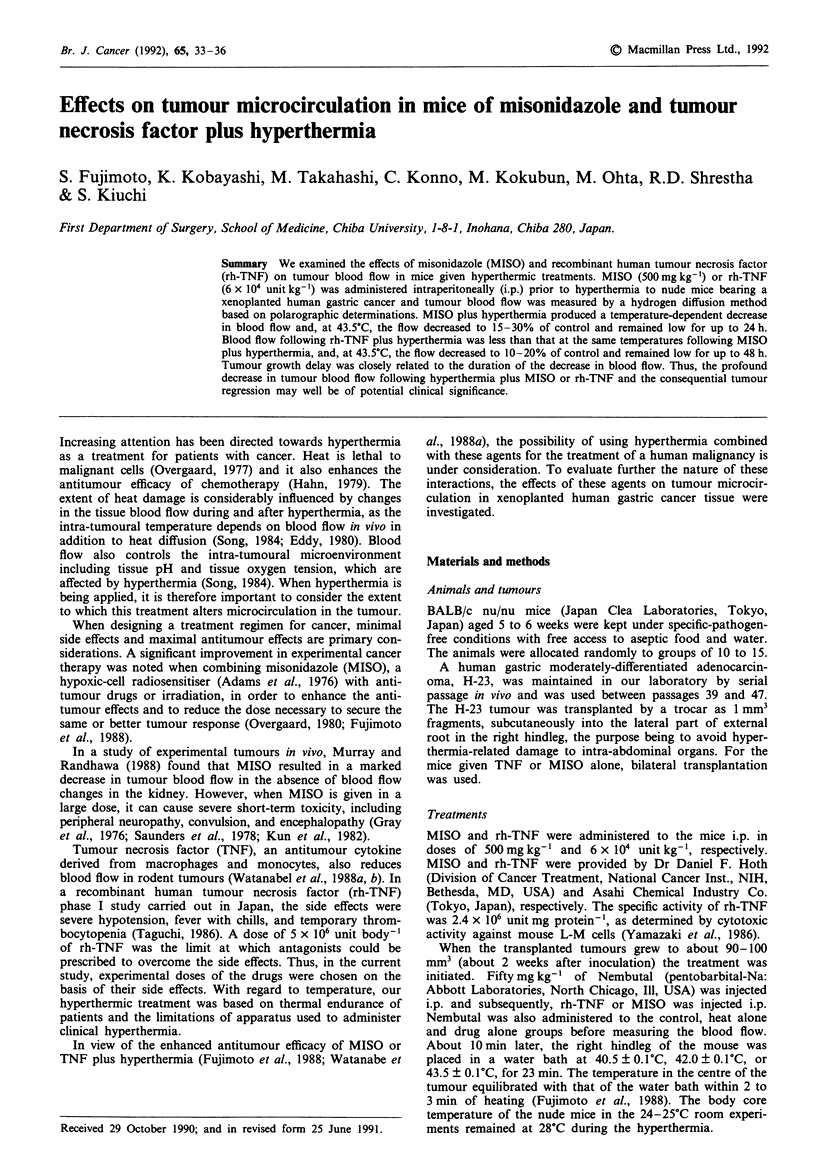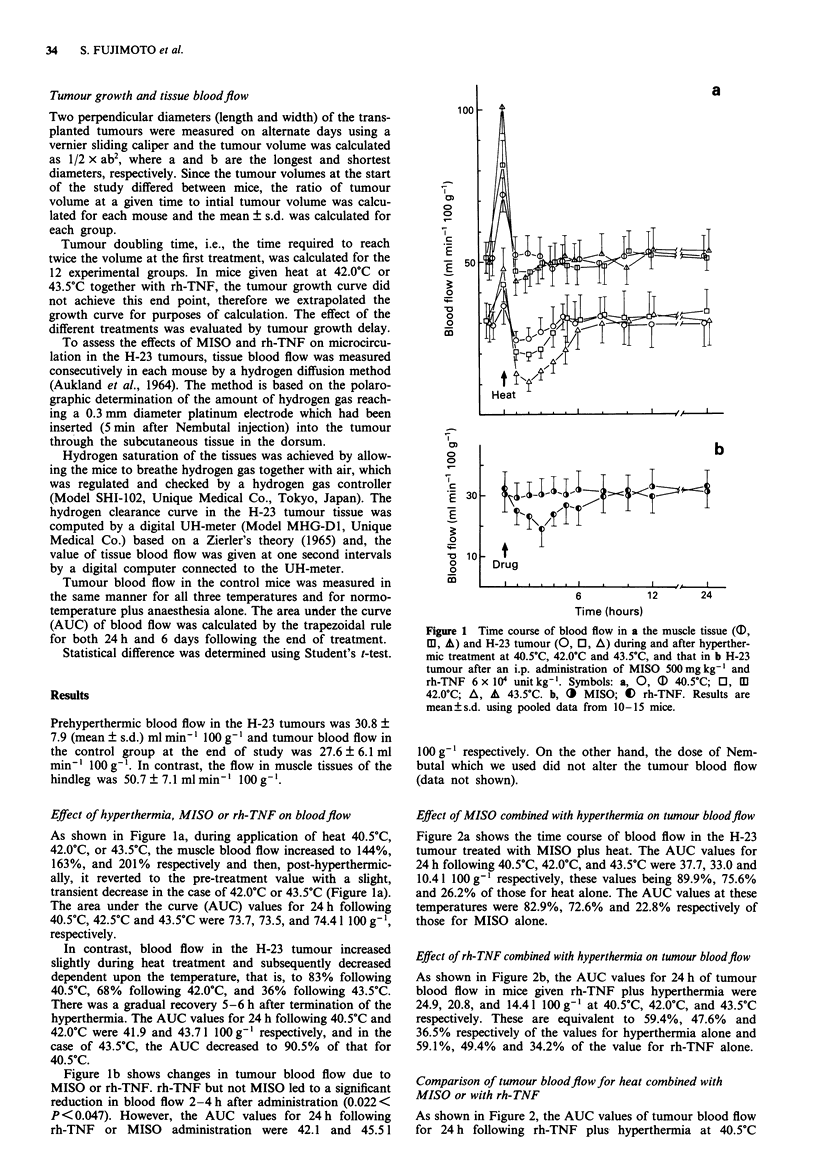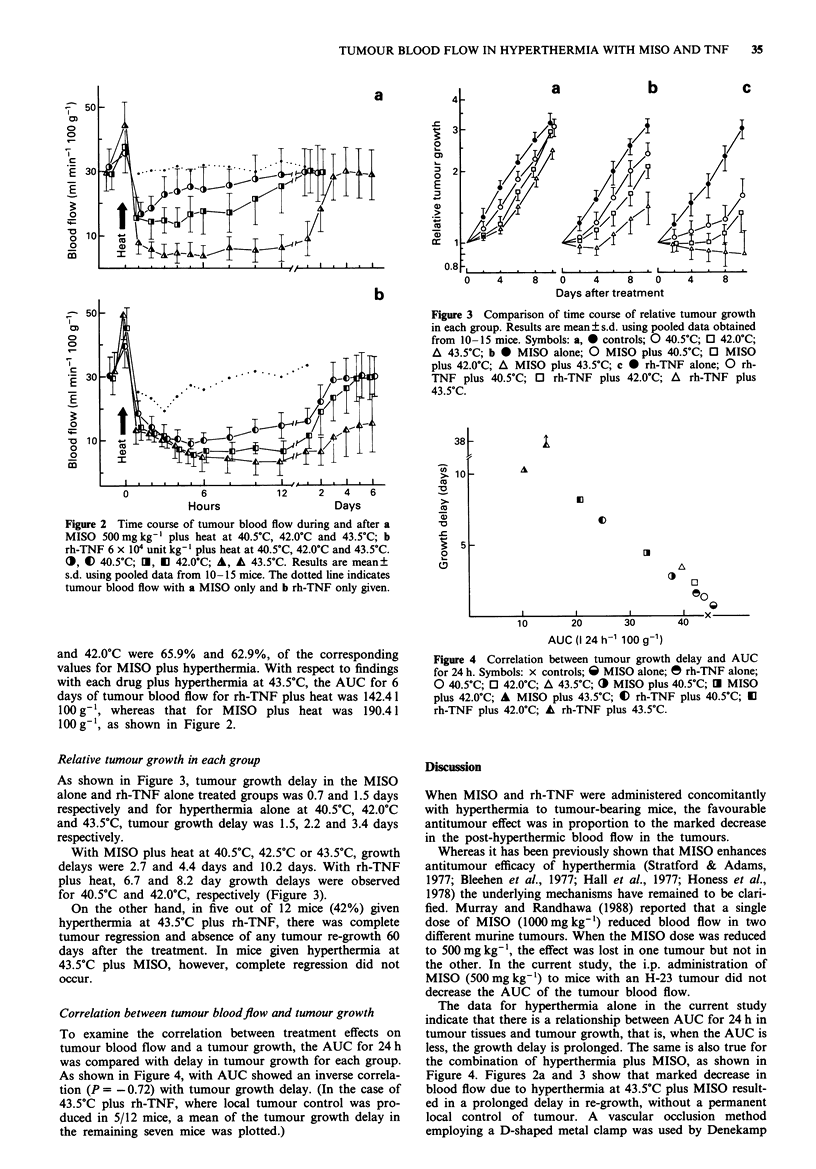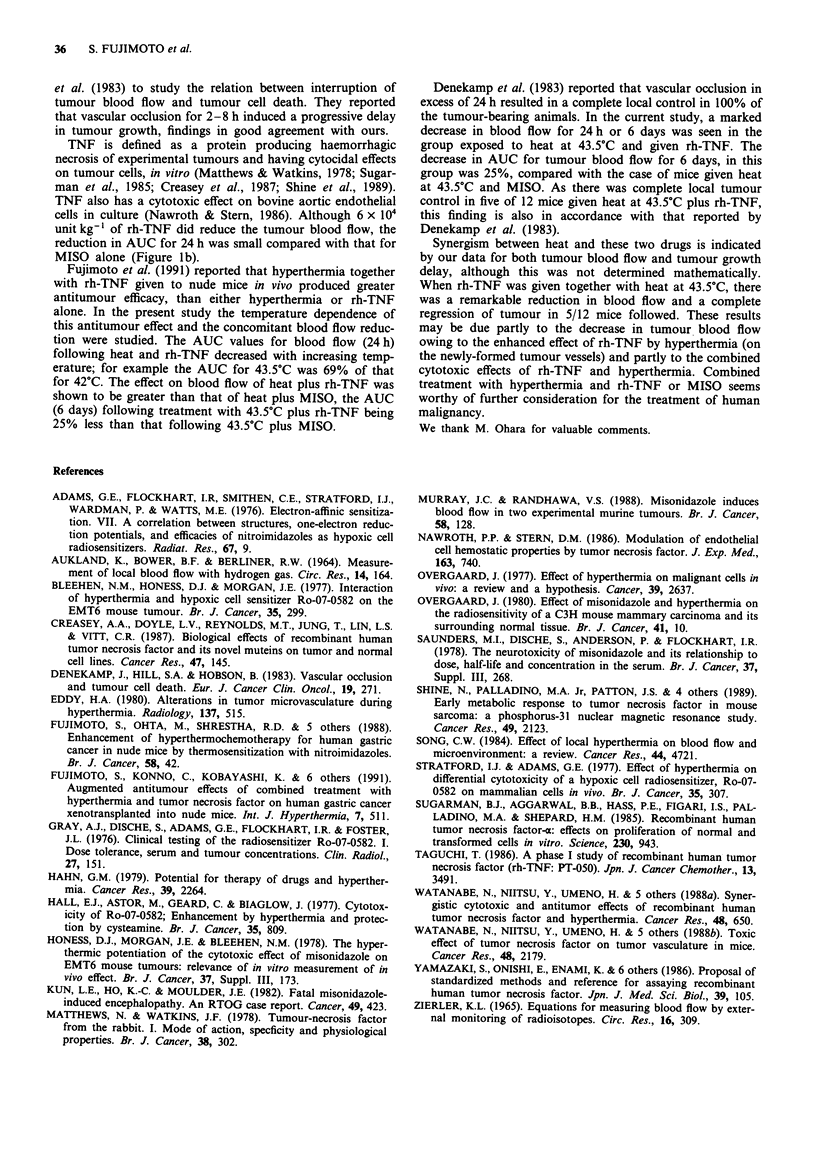# Effects on tumour microcirculation in mice of misonidazole and tumour necrosis factor plus hyperthermia.

**DOI:** 10.1038/bjc.1992.6

**Published:** 1992-01

**Authors:** S. Fujimoto, K. Kobayashi, M. Takahashi, C. Konno, M. Kokubun, M. Ohta, R. D. Shrestha, S. Kiuchi

**Affiliations:** First Department of Surgery, School of Medicine, Chiba University, Japan.

## Abstract

We examined the effects of misonidazole (MISO) and recombinant human tumour necrosis factor (rh-TNF) on tumour blood flow in mice given hyperthermic treatments. MISO (500 mg kg-1) or rh-TNF (6 x 10(4) unit kg-1) was administered intraperitoneally (i.p.) prior to hyperthermia to nude mice bearing a xenoplanted human gastric cancer and tumour blood flow was measured by a hydrogen diffusion method based on polarographic determinations. MISO plus hyperthermia produced a temperature-dependent decrease in blood flow and, at 43.5 degrees C, the flow decreased to 15-30% of control and remained low for up to 24 h. Blood flow following rh-TNF plus hyperthermia was less than that at the same temperatures following MISO plus hyperthermia, and, at 43.5 degrees C, the flow decreased to 10-20% of control and remained low for up to 48 h. Tumour growth delay was closely related to the duration of the decrease in blood flow. Thus, the profound decrease in tumour blood flow following hyperthermia plus MISO or rh-TNF and the consequential tumour regression may well be of potential clinical significance.


*Br. J. Cancer* (1992), **65**, 33–36 © Macmillan Press Ltd., 1992

### Effects on tumour microcirculation in mice of misonidazole and tumour necrosis factor plus hyperthermia

S. Fujimoto, K. Kobayashi, M. Takahashi, C. Konno, M. Kokubun, M. Ohta, R.D. Shrestha & S. Kiuchi

*First Department of Surgery, School of Medicine, Chiba University, 1-8-1, Inohana, Chiba 280, Japan.*

**Summary** We examined the effects of misonidazole (MISO) and recombinant human tumour necrosis factor (rh-TNF) on tumour blood flow in mice given hyperthermic treatments. MISO ($500\,mg\,kg^{-1}$) or rh-TNF ($6 \times 10^4$ unit $kg^{-1}$) was administered intraperitoneally (i.p.) prior to hyperthermia to nude mice bearing a xenoplanted human gastric cancer and tumour blood flow was measured by a hydrogen diffusion method based on polarographic determinations. MISO plus hyperthermia produced a temperature-dependent decrease in blood flow and, at 43.5°C, the flow decreased to 15–30% of control and remained low for up to 24 h. Blood flow following rh-TNF plus hyperthermia was less than that at the same temperatures following MISO plus hyperthermia, and, at 43.5°C, the flow decreased to 10–20% of control and remained low for up to 48 h. Tumour growth delay was closely related to the duration of the decrease in blood flow. Thus, the profound decrease in tumour blood flow following hyperthermia plus MISO or rh-TNF and the consequential tumour regression may well be of potential clinical significance.

Increasing attention has been directed towards hyperthermia as a treatment for patients with cancer. Heat is lethal to malignant cells (Overgaard, 1977) and it also enhances the antitumour efficacy of chemotherapy (Hahn, 1979). The extent of heat damage is considerably influenced by changes in the tissue blood flow during and after hyperthermia, as the intra-tumoural temperature depends on blood flow *in vivo* in addition to heat diffusion (Song, 1984; Eddy, 1980). Blood flow also controls the intra-tumoural microenvironment including tissue pH and tissue oxygen tension, which are affected by hyperthermia (Song, 1984). When hyperthermia is being applied, it is therefore important to consider the extent to which this treatment alters microcirculation in the tumour.

When designing a treatment regimen for cancer, minimal side effects and maximal antitumour effects are primary considerations. A significant improvement in experimental cancer therapy was noted when combining misonidazole (MISO), a hypoxic-cell radiosensitiser (Adams *et al.*, 1976) with antitumour drugs or irradiation, in order to enhance the antitumour effects and to reduce the dose necessary to secure the same or better tumour response (Overgaard, 1980; Fujimoto *et al.*, 1988).

In a study of experimental tumours *in vivo*, Murray and Randhawa (1988) found that MISO resulted in a marked decrease in tumour blood flow in the absence of blood flow changes in the kidney. However, when MISO is given in a large dose, it can cause severe short-term toxicity, including peripheral neuropathy, convulsion, and encephalopathy (Gray *et al.*, 1976; Saunders *et al.*, 1978; Kun *et al.*, 1982).

Tumour necrosis factor (TNF), an antitumour cytokine derived from macrophages and monocytes, also reduces blood flow in rodent tumours (Watanabel *et al.*, 1988*a*, *b*). In a recombinant human tumour necrosis factor (rh-TNF) phase I study carried out in Japan, the side effects were severe hypotension, fever with chills, and temporary thrombocytopenia (Taguchi, 1986). A dose of $5 \times 10^6$ unit $body^{-1}$ of rh-TNF was the limit at which antagonists could be prescribed to overcome the side effects. Thus, in the current study, experimental doses of the drugs were chosen on the basis of their side effects. With regard to temperature, our hyperthermic treatment was based on thermal endurance of patients and the limitations of apparatus used to administer clinical hyperthermia.

In view of the enhanced antitumour efficacy of MISO or TNF plus hyperthermia (Fujimoto *et al.*, 1988; Watanabe *et al.*, 1988*a*), the possibility of using hyperthermia combined with these agents for the treatment of a human malignancy is under consideration. To evaluate further the nature of these interactions, the effects of these agents on tumour microcirculation in xenoplanted human gastric cancer tissue were investigated.

Received 29 October 1990; and in revised form 25 June 1991.

### Materials and methods

### *Animals and tumours*

BALB/c nu/nu mice (Japan Clea Laboratories, Tokyo, Japan) aged 5 to 6 weeks were kept under specific-pathogen-free conditions with free access to aseptic food and water. The animals were allocated randomly to groups of 10 to 15.

A human gastric moderately-differentiated adenocarcinoma, H-23, was maintained in our laboratory by serial passage *in vivo* and was used between passages 39 and 47. The H-23 tumour was transplanted by a trocar as $1\,mm^3$ fragments, subcutaneously into the lateral part of external root in the right hindleg, the purpose being to avoid hyperthermia-related damage to intra-abdominal organs. For the mice given TNF or MISO alone, bilateral transplantation was used.

### *Treatments*

MISO and rh-TNF were administered to the mice i.p. in doses of $500\,mg\,kg^{-1}$ and $6 \times 10^4$ unit $kg^{-1}$, respectively. MISO and rh-TNF were provided by Dr Daniel F. Hoth (Division of Cancer Treatment, National Cancer Inst., NIH, Bethesda, MD, USA) and Asahi Chemical Industry Co. (Tokyo, Japan), respectively. The specific activity of rh-TNF was $2.4 \times 10^6$ unit mg $protein^{-1}$, as determined by cytotoxic activity against mouse L-M cells (Yamazaki *et al.*, 1986).

When the transplanted tumours grew to about 90–100 $mm^3$ (about 2 weeks after inoculation) the treatment was initiated. Fifty $mg\,kg^{-1}$ of Nembutal (pentobarbital-Na: Abbott Laboratories, North Chicago, Ill, USA) was injected i.p. and subsequently, rh-TNF or MISO was injected i.p. Nembutal was also administered to the control, heat alone and drug alone groups before measuring the blood flow. About 10 min later, the right hindleg of the mouse was placed in a water bath at $40.5 \pm 0.1$°C, $42.0 \pm 0.1$°C, or $43.5 \pm 0.1$°C, for 23 min. The temperature in the centre of the tumour equilibrated with that of the water bath within 2 to 3 min of heating (Fujimoto *et al.*, 1988). The body core temperature of the nude mice in the 24–25°C room experiments remained at 28°C during the hyperthermia.

*Tumour growth and tissue blood flow*

Two perpendicular diameters (length and width) of the transplanted tumours were measured on alternate days using a vernier sliding caliper and the tumour volume was calculated as $1/2 \times ab^2$, where a and b are the longest and shortest diameters, respectively. Since the tumour volumes at the start of the study differed between mice, the ratio of tumour volume at a given time to intial tumour volume was calculated for each mouse and the mean ± s.d. was calculated for each group.

Tumour doubling time, i.e., the time required to reach twice the volume at the first treatment, was calculated for the 12 experimental groups. In mice given heat at 42.0°C or 43.5°C together with rh-TNF, the tumour growth curve did not achieve this end point, therefore we extrapolated the growth curve for purposes of calculation. The effect of the different treatments was evaluated by tumour growth delay.

To assess the effects of MISO and rh-TNF on microcirculation in the H-23 tumours, tissue blood flow was measured consecutively in each mouse by a hydrogen diffusion method (Aukland *et al.*, 1964). The method is based on the polarographic determination of the amount of hydrogen gas reaching a 0.3 mm diameter platinum electrode which had been inserted (5 min after Nembutal injection) into the tumour through the subcutaneous tissue in the dorsum.

Hydrogen saturation of the tissues was achieved by allowing the mice to breathe hydrogen gas together with air, which was regulated and checked by a hydrogen gas controller (Model SHI-102, Unique Medical Co., Tokyo, Japan). The hydrogen clearance curve in the H-23 tumour tissue was computed by a digital UH-meter (Model MHG-D1, Unique Medical Co.) based on a Zierler's theory (1965) and, the value of tissue blood flow was given at one second intervals by a digital computer connected to the UH-meter.

Tumour blood flow in the control mice was measured in the same manner for all three temperatures and for normotemperature plus anaesthesia alone. The area under the curve (AUC) of blood flow was calculated by the trapezoidal rule for both 24 h and 6 days following the end of treatment.

Statistical difference was determined using Student's *t*-test.

### Results

Prehyperthermic blood flow in the H-23 tumours was 30.8 ± 7.9 (mean ± s.d.) ml $min^{-1}$ 100 $g^{-1}$ and tumour blood flow in the control group at the end of study was 27.6 ± 6.1 ml $min^{-1}$ 100 $g^{-1}$. In contrast, the flow in muscle tissues of the hindleg was 50.7 ± 7.1 ml $min^{-1}$ 100 $g^{-1}$.

**Figure 1** Time course of blood flow in **a** the muscle tissue (◐, ◫, ▲) and H-23 tumour (○, □, △) during and after hyperthermic treatment at 40.5°C, 42.0°C and 43.5°C, and that in **b** H-23 tumour after an i.p. administration of MISO 500 mg $kg^{-1}$ and rh-TNF $6 \times 10^4$ unit $kg^{-1}$. Symbols: **a**, ○, ◐ 40.5°C; □, ◫ 42.0°C; △, ▲ 43.5°C. **b**, ◑ MISO; ◐ rh-TNF. Results are mean±s.d. using pooled data from 10–15 mice.

*Effect of hyperthermia, MISO or rh-TNF on blood flow*

As shown in Figure 1a, during application of heat 40.5°C, 42.0°C, or 43.5°C, the muscle blood flow increased to 144%, 163%, and 201% respectively and then, post-hyperthermically, it reverted to the pre-treatment value with a slight, transient decrease in the case of 42.0°C or 43.5°C (Figure 1a). The area under the curve (AUC) values for 24 h following 40.5°C, 42.5°C and 43.5°C were 73.7, 73.5, and 74.4 l 100 $g^{-1}$, respectively.

In contrast, blood flow in the H-23 tumour increased slightly during heat treatment and subsequently decreased dependent upon the temperature, that is, to 83% following 40.5°C, 68% following 42.0°C, and 36% following 43.5°C. There was a gradual recovery 5–6 h after termination of the hyperthermia. The AUC values for 24 h following 40.5°C and 42.0°C were 41.9 and 43.7 l 100 $g^{-1}$ respectively, and in the case of 43.5°C, the AUC decreased to 90.5% of that for 40.5°C.

Figure 1b shows changes in tumour blood flow due to MISO or rh-TNF. rh-TNF but not MISO led to a significant reduction in blood flow 2–4 h after administration ($0.022 < P < 0.047$). However, the AUC values for 24 h following rh-TNF or MISO administration were 42.1 and 45.5 l 100 $g^{-1}$ respectively. On the other hand, the dose of Nembutal which we used did not alter the tumour blood flow (data not shown).

*Effect of MISO combined with hyperthermia on tumour blood flow*

Figure 2a shows the time course of blood flow in the H-23 tumour treated with MISO plus heat. The AUC values for 24 h following 40.5°C, 42.0°C, and 43.5°C were 37.7, 33.0 and 10.4 l 100 $g^{-1}$ respectively, these values being 89.9%, 75.6% and 26.2% of those for heat alone. The AUC values at these temperatures were 82.9%, 72.6% and 22.8% respectively of those for MISO alone.

*Effect of rh-TNF combined with hyperthermia on tumour blood flow*

As shown in Figure 2b, the AUC values for 24 h of tumour blood flow in mice given rh-TNF plus hyperthermia were 24.9, 20.8, and 14.4 l 100 $g^{-1}$ at 40.5°C, 42.0°C, and 43.5°C respectively. These are equivalent to 59.4%, 47.6% and 36.5% respectively of the values for hyperthermia alone and 59.1%, 49.4% and 34.2% of the value for rh-TNF alone.

*Comparison of tumour blood flow for heat combined with MISO or with rh-TNF*

As shown in Figure 2, the AUC values of tumour blood flow for 24 h following rh-TNF plus hyperthermia at 40.5°C

Figure 2 Time course of tumour blood flow during and after a MISO 500 mg kg$^{-1}$ plus heat at 40.5°C, 42.0°C and 43.5°C; b rh-TNF 6 × 10$^{4}$ unit kg$^{-1}$ plus heat at 40.5°C, 42.0°C and 43.5°C. ◑, ◐ 40.5°C; ◨, ◧ 42.0°C; ▲, ▲ 43.5°C. Results are mean ± s.d. using pooled data from 10–15 mice. The dotted line indicates tumour blood flow with a MISO only and b rh-TNF only given.

Figure 3 Comparison of time course of relative tumour growth in each group. Results are mean ± s.d. using pooled data obtained from 10–15 mice. Symbols: a, ● controls; ○ 40.5°C; □ 42.0°C; △ 43.5°C; b ● MISO alone; ○ MISO plus 40.5°C; □ MISO plus 42.0°C; △ MISO plus 43.5°C; c ● rh-TNF alone; ○ rh-TNF plus 40.5°C; □ rh-TNF plus 42.0°C; △ rh-TNF plus 43.5°C.

Figure 4 Correlation between tumour growth delay and AUC for 24 h. Symbols: × controls; ⊖ MISO alone; ⊖ rh-TNF alone; ○ 40.5°C; □ 42.0°C; △ 43.5°C; ◑ MISO plus 40.5°C; ◨ MISO plus 42.0°C; ▲ MISO plus 43.5°C; ◐ rh-TNF plus 40.5°C; ◧ rh-TNF plus 42.0°C; ▲ rh-TNF plus 43.5°C.

and 42.0°C were 65.9% and 62.9%, of the corresponding values for MISO plus hyperthermia. With respect to findings with each drug plus hyperthermia at 43.5°C, the AUC for 6 days of tumour blood flow for rh-TNF plus heat was 142.4 l 100 g$^{-1}$, whereas that for MISO plus heat was 190.4 l 100 g$^{-1}$, as shown in Figure 2.

*Relative tumour growth in each group*

As shown in Figure 3, tumour growth delay in the MISO alone and rh-TNF alone treated groups was 0.7 and 1.5 days respectively and for hyperthermia alone at 40.5°C, 42.0°C and 43.5°C, tumour growth delay was 1.5, 2.2 and 3.4 days respectively.

With MISO plus heat at 40.5°C, 42.5°C or 43.5°C, growth delays were 2.7 and 4.4 days and 10.2 days. With rh-TNF plus heat, 6.7 and 8.2 day growth delays were observed for 40.5°C and 42.0°C, respectively (Figure 3).

On the other hand, in five out of 12 mice (42%) given hyperthermia at 43.5°C plus rh-TNF, there was complete tumour regression and absence of any tumour re-growth 60 days after the treatment. In mice given hyperthermia at 43.5°C plus MISO, however, complete regression did not occur.

*Correlation between tumour bloodflow and tumour growth*

To examine the correlation between treatment effects on tumour blood flow and a tumour growth, the AUC for 24 h was compared with delay in tumour growth for each group. As shown in Figure 4, with AUC showed an inverse correlation ($P = -0.72$) with tumour growth delay. (In the case of 43.5°C plus rh-TNF, where local tumour control was produced in 5/12 mice, a mean of the tumour growth delay in the remaining seven mice was plotted.)

### Discussion

When MISO and rh-TNF were administered concomitantly with hyperthermia to tumour-bearing mice, the favourable antitumour effect was in proportion to the marked decrease in the post-hyperthermic blood flow in the tumours.

Whereas it has been previously shown that MISO enhances antitumour efficacy of hyperthermia (Stratford & Adams, 1977; Bleehen *et al.*, 1977; Hall *et al.*, 1977; Honess *et al.*, 1978) the underlying mechanisms have remained to be clarified. Murray and Randhawa (1988) reported that a single dose of MISO (1000 mg kg$^{-1}$) reduced blood flow in two different murine tumours. When the MISO dose was reduced to 500 mg kg$^{-1}$, the effect was lost in one tumour but not in the other. In the current study, the i.p. administration of MISO (500 mg kg$^{-1}$) to mice with an H-23 tumour did not decrease the AUC of the tumour blood flow.

The data for hyperthermia alone in the current study indicate that there is a relationship between AUC for 24 h in tumour tissues and tumour growth, that is, when the AUC is less, the growth delay is prolonged. The same is also true for the combination of hyperthermia plus MISO, as shown in Figure 4. Figures 2a and 3 show that marked decrease in blood flow due to hyperthermia at 43.5°C plus MISO resulted in a prolonged delay in re-growth, without a permanent local control of tumour. A vascular occlusion method employing a D-shaped metal clamp was used by Denekamp et al. (1983) to study the relation between interruption of tumour blood flow and tumour cell death. They reported that vascular occlusion for 2–8 h induced a progressive delay in tumour growth, findings in good agreement with ours.

TNF is defined as a protein producing haemorrhagic necrosis of experimental tumours and having cytocidal effects on tumour cells, *in vitro* (Matthews & Watkins, 1978; Sugarman *et al.*, 1985; Creasey *et al.*, 1987; Shine *et al.*, 1989). TNF also has a cytotoxic effect on bovine aortic endothelial cells in culture (Nawroth & Stern, 1986). Although $6 \times 10^4$ unit $kg^{-1}$ of rh-TNF did reduce the tumour blood flow, the reduction in AUC for 24 h was small compared with that for MISO alone (Figure 1b).

Fujimoto *et al.* (1991) reported that hyperthermia together with rh-TNF given to nude mice *in vivo* produced greater antitumour efficacy, than either hyperthermia or rh-TNF alone. In the present study the temperature dependence of this antitumour effect and the concomitant blood flow reduction were studied. The AUC values for blood flow (24 h) following heat and rh-TNF decreased with increasing temperature; for example the AUC for 43.5°C was 69% of that for 42°C. The effect on blood flow of heat plus rh-TNF was shown to be greater than that of heat plus MISO, the AUC (6 days) following treatment with 43.5°C plus rh-TNF being 25% less than that following 43.5°C plus MISO.

Denekamp *et al.* (1983) reported that vascular occlusion in excess of 24 h resulted in a complete local control in 100% of the tumour-bearing animals. In the current study, a marked decrease in blood flow for 24 h or 6 days was seen in the group exposed to heat at 43.5°C and given rh-TNF. The decrease in AUC for tumour blood flow for 6 days, in this group was 25%, compared with the case of mice given heat at 43.5°C and MISO. As there was complete local tumour control in five of 12 mice given heat at 43.5°C plus rh-TNF, this finding is also in accordance with that reported by Denekamp *et al.* (1983).

Synergism between heat and these two drugs is indicated by our data for both tumour blood flow and tumour growth delay, although this was not determined mathematically. When rh-TNF was given together with heat at 43.5°C, there was a remarkable reduction in blood flow and a complete regression of tumour in 5/12 mice followed. These results may be due partly to the decrease in tumour blood flow owing to the enhanced effect of rh-TNF by hyperthermia (on the newly-formed tumour vessels) and partly to the combined cytotoxic effects of rh-TNF and hyperthermia. Combined treatment with hyperthermia and rh-TNF or MISO seems worthy of further consideration for the treatment of human malignancy.

We thank M. Ohara for valuable comments.

### References

ADAMS, G.E., FLOCKHART, I.R, SMITHEN, C.E., STRATFORD, I.J., WARDMAN, P. & WATTS, M.E. (1976). Electron-affinic sensitization. VII. A correlation between structures, one-electron reduction potentials, and efficacies of nitroimidazoles as hypoxic cell radiosensitizers. *Radiat. Res.*, **67**, 9.

AUKLAND, K., BOWER, B.F. & BERLINER, R.W. (1964). Measurement of local blood flow with hydrogen gas. *Circ. Res.*, **14**, 164.

BLEEHEN, N.M., HONESS, D.J. & MORGAN, J.E. (1977). Interaction of hyperthermia and hypoxic cell sensitizer Ro-07-0582 on the EMT6 mouse tumour. *Br. J. Cancer*, **35**, 299.

CREASEY, A.A., DOYLE, L.V., REYNOLDS, M.T., JUNG, T., LIN, L.S. & VITT, C.R. (1987). Biological effects of recombinant human tumor necrosis factor and its novel muteins on tumor and normal cell lines. *Cancer Res.*, **47**, 145.

DENEKAMP, J., HILL, S.A. & HOBSON, B. (1983). Vascular occlusion and tumour cell death. *Eur. J. Cancer Clin. Oncol.*, **19**, 271.

EDDY, H.A. (1980). Alterations in tumor microvasculature during hyperthermia. *Radiology*, **137**, 515.

FUJIMOTO, S., OHTA, M., SHRESTHA, R.D. & 5 others (1988). Enhancement of hyperthermochemotherapy for human gastric cancer in nude mice by thermosensitization with nitroimidazoles. *Br. J. Cancer*, **58**, 42.

FUJIMOTO, S., KONNO, C., KOBAYASHI, K. & 6 others (1991). Augmented antitumour effects of combined treatment with hyperthermia and tumor necrosis factor on human gastric cancer xenotransplanted into nude mice. *Int. J. Hyperthermia*, **7**, 511.

GRAY, A.J., DISCHE, S., ADAMS, G.E., FLOCKHART, I.R. & FOSTER, J.L. (1976). Clinical testing of the radiosensitizer Ro-07-0582. I. Dose tolerance, serum and tumour concentrations. *Clin. Radiol.*, **27**, 151.

HAHN, G.M. (1979). Potential for therapy of drugs and hyperthermia. *Cancer Res.*, **39**, 2264.

HALL, E.J., ASTOR, M., GEARD, C. & BIAGLOW, J. (1977). Cytotoxicity of Ro-07-0582; Enhancement by hyperthermia and protection by cysteamine. *Br. J. Cancer*, **35**, 809.

HONESS, D.J., MORGAN, J.E. & BLEEHEN, N.M. (1978). The hyperthermic potentiation of the cytotoxic effect of misonidazole on EMT6 mouse tumours: relevance of *in vitro* measurement of *in vivo* effect. *Br. J. Cancer*, **37**, Suppl. III, 173.

KUN, L.E., HO, K.-C. & MOULDER, J.E. (1982). Fatal misonidazole-induced encephalopathy. An RTOG case report. *Cancer*, **49**, 423.

MATTHEWS, N. & WATKINS, J.F. (1978). Tumour-necrosis factor from the rabbit. I. Mode of action, specficity and physiological properties. *Br. J. Cancer*, **38**, 302.

MURRAY, J.C. & RANDHAWA, V.S. (1988). Misonidazole induces blood flow in two experimental murine tumours. *Br. J. Cancer*, **58**, 128.

NAWROTH, P.P. & STERN, D.M. (1986). Modulation of endothelial cell hemostatic properties by tumor necrosis factor. *J. Exp. Med.*, **163**, 740.

OVERGAARD, J. (1977). Effect of hyperthermia on malignant cells *in vivo*: a review and a hypothesis. *Cancer*, **39**, 2637.

OVERGAARD, J. (1980). Effect of misonidazole and hyperthermia on the radiosensitivity of a C3H mouse mammary carcinoma and its surrounding normal tissue. *Br. J. Cancer*, **41**, 10.

SAUNDERS, M.I., DISCHE, S., ANDERSON, P. & FLOCKHART, I.R. (1978). The neurotoxicity of misonidazole and its relationship to dose, half-life and concentration in the serum. *Br. J. Cancer*, **37**, Suppl. III, 268.

SHINE, N., PALLADINO, M.A. Jr, PATTON, J.S. & 4 others (1989). Early metabolic response to tumor necrosis factor in mouse sarcoma: a phosphorus-31 nuclear magnetic resonance study. *Cancer Res.*, **49**, 2123.

SONG, C.W. (1984). Effect of local hyperthermia on blood flow and microenvironment: a review. *Cancer Res.*, **44**, 4721.

STRATFORD, I.J. & ADAMS, G.E. (1977). Effect of hyperthermia on differential cytotoxicity of a hypoxic cell radiosensitizer, Ro-07-0582 on mammalian cells *in vivo*. *Br. J. Cancer*, **35**, 307.

SUGARMAN, B.J., AGGARWAL, B.B., HASS, P.E., FIGARI, I.S., PALLADINO, M.A. & SHEPARD, H.M. (1985). Recombinant human tumor necrosis factor-α: effects on proliferation of normal and transformed cells *in vitro*. *Science*, **230**, 943.

TAGUCHI, T. (1986). A phase I study of recombinant human tumor necrosis factor (rh-TNF: PT-050). *Jpn. J. Cancer Chemother.*, **13**, 3491.

WATANABE, N., NIITSU, Y., UMENO, H. & 5 others (1988*a*). Synergistic cytotoxic and antitumor effects of recombinant human tumor necrosis factor and hyperthermia. *Cancer Res.*, **48**, 650.

WATANABE, N., NIITSU, Y., UMENO, H. & 5 others (1988*b*). Toxic effect of tumor necrosis factor on tumor vasculature in mice. *Cancer Res.*, **48**, 2179.

YAMAZAKI, S., ONISHI, E., ENAMI, K. & 6 others (1986). Proposal of standardized methods and reference for assaying recombinant human tumor necrosis factor. *Jpn. J. Med. Sci. Biol.*, **39**, 105.

ZIERLER, K.L. (1965). Equations for measuring blood flow by external monitoring of radioisotopes. *Circ. Res.*, **16**, 309.